# Assessing Daily Function and Sleep Disorders in Hemodialysis Patients with End-Stage Renal Disease

**DOI:** 10.3390/healthcare12212115

**Published:** 2024-10-23

**Authors:** Maria Saridi, Georgios Batziogiorgos, Aikaterini Toska, Ioanna Dimitriadou, Foteini Malli, Stella Zetta, Evangelos C. Fradelos

**Affiliations:** 1 Laboratory of Clinical Nursing, Department of Nursing, University of Thessaly, 41500 Larissa, Greece; saridi@uth.gr (M.S.); ktoska@uth.gr (A.T.); ioadimitriadou@uth.gr (I.D.); szetta@uth.gr (S.Z.); 2 Infection Control Unit, General Hospital of Trikala, 42131 Trikala, Greece; batziogiorgos@yahoo.gr; 3 Respiratory Disorders Laboratory, Faculty of Nursing, University of Thessaly, 41110 Larissa, Greece; fmalli@uth.gr

**Keywords:** hemodialysis patients, renal failure, daily activities, sleep disorders

## Abstract

Background: Chronic, end-stage renal disease significantly impacts patients’ daily activities and sleep quality, particularly those undergoing hemodialysis. However, there is limited research on the extent of these challenges and their correlation with this population. Aim: This study aims to assess the level of activity of daily living and the prevalence of sleep disturbances in patients with end-stage renal disease undergoing hemodialysis. Method: A cross-sectional study involved 130 patients receiving hemodialysis in two public General Hospitals in Greece. The Barthel Index was used to measure daily living activity, while the Athens Insomnia Scale assessed sleep disorders. The data were analyzed using SPSSV25.0. Results: Of the 210 questionnaires, 130 were returned fully completed (response rate 62%). Most of the sample participants suffered from comorbidities (76.9%). The total Barthel Index score showed moderate dependence for patients, significantly related to the years and hours of dialysis (*p* = 0.007 and *p* = 0.000, respectively). The total score of the Athens Insomnia Scale was also significantly associated with age (*p* = 0.029), marital status (*p* = 0.015) and the years and hours of hemodialysis (*p* = 0.004 and *p* = 0.001, respectively). A statistically significant difference was recorded between the daily activity of patients with end-stage renal failure and their sleep quality (*p* = 0.000) Finally, the physical activity level of the participants was related to the existence of another physical health (*p* = 0.000) or mental health problem (*p* = 0.000). Conclusions: Hemodialysis patients with chronic, end-stage renal disease experience significant challenges in maintaining daily activities and are prone to sleep disorders. These findings suggest a need for integrated care strategies that address both physical function and sleep quality to improve the overall well-being of this population.

## 1. Introduction

Chronic kidney disease (CKD) is a growing global public health priority associated with very high morbidity and mortality, causing a significant burden to health systems. In 2017, a total of 850 million people were estimated to be living with CKD, which was twice the estimated global prevalence of diabetes and more than 20 times the estimated global prevalence of HIV [1].

In 2020, the incidence of CKD was 12 per million population among people aged 0–17, 118 among people aged 18–44, 598 among people aged 45–64, 1225 among people aged 65–74, and 1447 among persons aged ≥75, with the data demonstrating an increase in the incidence with increasing age [2].

Hemodialysis is a necessary, but perhaps aggressive, intervention for people who have lost their kidney function, which must be repeated almost every two days, with the need to travel from the patient’s home to the dialysis center, carrying a burden of time and money both for the patient and their caregivers [3,4]. Over the past sixty years, hemodialysis has been the most popular method of renal replacement, with accessibility and availability varying worldwide, with nearly four million people worldwide undergoing some form of renal replacement therapy, with hemodialysis being the most popular [2].

This procedure can cause various effects for the patient, such as feeling tired after the session, nausea, dizziness, fainting, a feeling of heaviness in the chest, a psycho-emotional burden, depression, financial and work difficulties, etc., which negatively affect their quality of life and their daily activities, and, therefore, they need specialized nursing care both physically, mentally, and spiritually [5,6,7,8]. Cardiovascular disease also affects over two-thirds of people undergoing hemodialysis and is the leading cause of morbidity, accounting for 50% of mortality in these patients, which is more than in the general population [2]. This overall decline in hemodialysis patients is especially aggravated in the period after hemodialysis, and the functionality they lose concerns simple procedures such as bathing, moving, climbing stairs, and dressing [9,10].

Dialysis patients often perceive that they remain passive participants throughout the whole spectrum of hemodialysis, from the decision to initiate the treatment, throughout the clinical implementation procedures, to the management of medication and dietary therapy. Nevertheless, serious weakness is caused by dialysis in daily practice, often rendering simple procedures challenging, resulting in a strong dependence on a caregiver. Added to this is the increased age of most dialysis patients [11]. However, in contemporary care, there is a tendency for these patients to be involved in decision-making procedures and to be encouraged to have self-care and autonomy in their daily lives [12,13]. Promoting the daily activity of people undergoing hemodialysis is an important approach for improving the quality of life and biological parameters, such as cardiovascular function, and reducing mortality [14,15].

Another impairment of daily functioning that is common in hemodialysis patients is sleep disorders [16]. A systematic review of 17 studies in patients undergoing hemodialysis showed that sleep disturbance was one of their most common symptoms, with an average prevalence of 44%, often missed by both the physicians and the nurses caring for these patients [17,18].

The quality of sleep is a key factor that can increase the fatigue and anxiety felt by the patient, weaken their memory, and bring about various changes in their behavior and psyche, but poor sleep quality also increases the patient’s risk of mortality, especially if it changes the nocturnal blood pressure [16,19,20,21]. Also, sleep disorders can affect the patient’s compliance with the treatment and medication and even lead to missed dialysis sessions [22,23].

Studies linking the relationship between sleep disorders and physical activity in hemodialysis patients show a significant improvement in their quality of life and mortality. Still, there is no extensive research on the association of daily physical activity with sleep disorders in these patients. Studies that have also investigated the daily activities of hemodialysis patients show that, in several cases, these patients can perform daily activities without assistance, with most of the difficulties being observed in walking and climbing the stairs. At the same time, an important intervention in dialysis clinics is strengthening daily physical activity with special programs to activate and mobilize these patients. Therefore, it is important to investigate the daily physical activity of these patients to identify their needs and to design a holistic care plan to strengthen individuals in their daily practice and improve their quality of life [24,25,26,27].

Thus, this study aims to fill this gap by investigating the levels of activity of daily living and the prevalence of sleep disorders in patients with chronic, end-stage renal disease undergoing hemodialysis.

## 2. Materials and Methods

### 2.1. Study Design and Setting

This cross-sectional study was conducted at the Artificial Kidney Units of two public General Hospitals (Trikala General Hospital and Karditsa General Hospital), in the region of Thessaly, involving patients with chronic, end-stage renal disease who were undergoing maintenance hemodialysis. The study period extended from August 2023 to October 2023.

### 2.2. Participants

The study included 130 adult patients who were diagnosed with end-stage renal disease and receiving regular hemodialysis treatment (210 questionnaires were distributed and 130 were returned, a 62% response rate). The participants were selected using a simple random sampling method. The inclusion criteria were an age ≥18 years, a diagnosis of end-stage renal disease requiring hemodialysis for at least three months, and the ability to provide informed consent. The exclusion criteria included patients with acute illnesses, those undergoing peritoneal dialysis, or those with cognitive impairments that could affect their ability to complete the assessment tools.

### 2.3. Data Collection Tools

The anonymous questionnaire that was used consisted of three parts.

A. Sociodemographics and clinical characteristics: This includes the participants’ demographics, such as their gender, age, place of residence, education level, marital status, and work status, as well as their clinical characteristics, such as their years of illness, years of hemodialysis, duration of hemodialysis, support from a regular caregiver, other physical problems (comorbidities), mental health problems, and medication uptake history.

B. Barthel Index questionnaire: The Barthel Index is used to investigate the level of daily activities and autonomy in chronic patients, such as dialysis patients [28,29,30]. The scale measures the performance of daily living activities, the functional independence level, and the mobility of the patient. This scale consists of ten questions that reflect the degree of activity according to the weighting; the Greek adaptation was used [31]. Items are rated in terms of whether individuals can perform activities independently, with some assistance, or are dependent, and they are allotted in the following way: 0 or 5 points per item for bathing and grooming; 0, 5, or 10 points per item for feeding, dressing, bowel control, bladder control, toilet use, and stairs; and 0, 5, 10, or 15 points per item for transfers and mobility. The Barthel Index yields a total score out of 100—the higher the score, the greater the degree of functional independence; more specifically, the scoring results are as follows: 0–20/complete dependence, 21–60/severe dependence, 61–90/moderate dependence, 91–99/mild dependence, and 100/complete independence. The scale shows excellent reliability (Cronbach α = 0.937).

C. Athens Insomnia Scale questionnaire: This scale records the participant’s assessment of the difficulties they may face with their sleep [32,33]. This questionnaire is a self-completion scale of eight items. The first five items assess their difficulty with sleep induction, awakenings during the night, morning awakening, total sleep time, and overall sleep quality, while the last three items address the consequences of insomnia that they face the following day (problems with their feeling of well-being, overall functionality, and sleepiness during the day). Each item of the questionnaire is rated 0, 1, 2, or 3, (with 0 corresponding to no problem and 3 to a very serious problem). Thus, the total score ranges from 0 (the absence of any sleep-related problem) to 24 (the most severe degree of insomnia). The participants were asked to rate each item positively (i.e., to choose between rating options 1, 2, and 3), only if they had experienced their sleep difficulty at least three times a week during the past month, i.e., a frequency that is consistent with ICD-10 criteria [34]. The Greek version of the questionnaire was used for this study. The scale shows excellent reliability (Cronbach α = 0.900).

### 2.4. Ethics

At all the stages of the study, all the rules of ethics were observed. Before conducting the study, the research protocol was approved by the scientific boards of the two hospitals. Regarding the patients, they signed their informed consent following the information being provided about the purpose of the research, the voluntary participation, and the possibility of withdrawing from the study if they requested it, at any stage of it. Their participation was voluntary, and throughout the study, the rules of confidentiality and anonymity were respected. The study received permission from the scientific councils of the two hospitals (No. Prot. 20322 24/7/2023 and No. Prot. 14191 26/7/2023).

### 2.5. Statistical Analysis

For the data analysis, the statistical package SPSSV25.0 (a statistical package for the social sciences) was used. Tables with frequencies, percentages, mean values, and standard deviations were used to present the results. The reliability of the questionnaires was measured with the Cronbach alpha index: for the Athens Insomnia Scale, we found α = 0.873, while for the Barthel Index, we found 0.90. Both of these values were >0.7, which shows high reliability. In the bivariate analysis, t-tests and an analysis of variance (for two or more categories of the independent variable, respectively) were used. Multivariate linear regression was used to examine if there are models that can predict the daily activity and sleep quality of CKD patients undergoing hemodialysis. To investigate this, two separate stepwise regression models were used, putting the Barthel Index total score as the dependent variable in model 1, and the Athens Insomnia Scale total score as the dependent variable in model 2. At the same time, all the sociodemographic and clinical characteristics with a *p*-value of <0.25 in the bivariate analysis were included in the multivariate analysis [35].

## 3. Results

### 3.1. Descriptive Analysis

The study involved 130 individuals undergoing extrarenal purification (hemodialysis), most of whom were men (60% vs. 40%), while 73.8% were over 60 years old and 80.8% had basic (mandatory) education. Detailed information regarding the patients’ demographic and clinical characteristics is presented in Table 1.

Concerning comorbidities, most of the patients in the sample (76.9%) reported that they face another health problem besides CKD/dialysis and receive pharmaceutical treatment for this disease as well. Finally, 13.1% of the patients reported that they face a mental health problem and are receiving medication for it.

### 3.2. Levels of Daily Living Activities/Barthel Index

The total Barthel Index score of 72.9 (±32.13) indicates moderate dependence for the patients, i.e., their daily living activity levels are considered good (see the Supplementary File). In addition, a bivariate analysis was performed to examine whether the total score of the Barthel Index was statistically significantly related to the demographic and clinical characteristics of the participants.

### 3.3. Sleep Difficulties/Athens Insomnia Scale Questionnaire

The total score of the Athens Insomnia Scale is 7.0154 (±4.72), which shows that the patients face a mild problem with sleep (see the Supplementary File).

### 3.4. Bivariate Analysis

#### 3.4.1. Association of Barthel Index with Clinical and Demographic Characteristics

The ANOVA test also showed that the total score of the Barthel Index was related to a statistically significant degree with the years that the patients were on dialysis (F(4,125) = 4.716, *p* = 0.007); more specifically, the patients who were on dialysis for more than 20 years had a mean value on the Barthel Index of 64.4, which was statistically significantly lower than those who were on dialysis for less than 5 years (86.6) (Bonferroni *p* < 0.001). In addition, the hours that the procedure lasted for were significantly related to the Barthel Index (F(3,125) = 9.842 *p* = 0.001); more specifically, the patients who were on dialysis for 2–3 h had a lower score, of 55.0, compared to those who were on dialysis for sessions of more than 4 h (Bonferroni *p* < 0.001). Also, a relationship was found with the existence of a regular caregiver (F(2,127) = 27.413 *p* = 0.000); more specifically, those who did not require a caregiver scored a 96 value, which was significantly higher than those who required informal caregivers, 71.4, and professional caregivers, 12.4 (Bonferroni *p* <0.001 in both cases). Finally, the independent sample t-test revealed that the level of physical activity of the participating patients was less strongly related to patients who were experiencing other health problems (value 66.3) compared to those with no additional health problems (value 94.7) (t(128) = 4.556 *p* = 0.001). Similarly, those who were experiencing mental health issues scored 21.7, a value that was significantly lower than those who were not experiencing mental health issues (80.6) (t(128) = 9.099, *p* = 0.000).

#### 3.4.2. Associations Between the Athens Insomnia Scale and the Clinical and Demographic Characteristics

Next, a bivariate analysis was performed to determine whether the total score of the Athens Insomnia Scale was statistically significantly related to the demographic and clinical characteristics of the participants. The ANOVA test showed that the total score of the Athens Insomnia Scale was related to a statistically significant degree with the age of the patients (F(4,125) = 2.785, *p* = 0.029); their marital status (F(4,128) = 4.715, *p* = 0.015), as the widowed participants scored a value of 8.2, which was significantly higher compared to the divorced participants (Bonferroni *p* = 0.05); the years that the patients had been on hemodialysis (F(4,125) = 3.590, *p* = 0.004), as the participants who had been on dialysis for 16–20 years scored a value of 12.8, which was significantly higher than those who were on dialysis for 6–10 years, who scored 5.5; the hours of hemodialysis (F(3,125) = 2.798, p = 0.001), with those who were on dialysis for sessions of less than 2 h reporting a 12.5 value, which was significantly higher compared to those who were in sessions for more than 4 h (Bonferroni *p* = 0.05); and the existence of a regular caregiver (F(2,127) = 10.732, *p* = 0.001). More specifically, those who did not require a caregiver scored a 3.3 value, which was significantly lower than those who required an informal caregiver (7.8) (Bonferroni *p* < 0.001) or a professional caregiver (8.4) (Bonferroni *p* = 0.48). Finally, the independent sample *t*-test (t(128) = 5.086, *p* = 0.000) revealed that the Athens Insomnia Scale score of the participating patients was related to the existence of other health problems (value 8.0), which was higher compared to those with no additional problem (value 3.5). Similarly, the independent sample *t*-test (t(128) = 4.556, *p* = 0.001) revealed a significant difference in the Athens Insomnia Scale score, as those who reported mental health issues had a score of 9.0, a value that was significantly higher than that of those who did not experience mental health issues, who reported a value of 6.7.

#### 3.4.3. Correlation of Physical Activity with Sleep Quality

Regarding the correlation between the daily activity levels and the sleep quality of the patients, the Pearson test was performed. A statistically significant difference (*p* = 0.000) was recorded between the physical activity of the patients with end-stage renal failure undergoing hemodialysis and their sleep quality. This correlation is negative (r = − 0.467/Sig. (2 − tailed) < 0.001) and indicates that as the level of physical activity of patients increases, sleep problems decrease.

### 3.5. Multiple Linear Regression Results

To investigate the associations between the demographic characteristics and the total score of the physical activity scales and sleep quality assessment, a multiple linear regression analysis was performed. The model was calculated to predict the activity level of the daily living of patients with end-stage renal failure who were undergoing hemodialysis and showed a result of R^2^ = 0.493, meaning that independent variables explained 49.3% of the variability of the dependent variable (Table 2).

In Table 2, a correlation was observed between the dependent variable (the Barthel Index) and the independent variables. It was found that the variables that participated in the regression model that was created and that affected the daily activity of the patients with end-stage renal failure who were undergoing hemodialysis were the years on dialysis, the presence of a caregiver, their work status, and insomnia.

The model evaluated the sleep characteristics of the CKD patients who were undergoing hemodialysis and showed that R^2^ = 0.219, predicting 21.9% of the variability of the dependent variable (Table 3). The model showed a correlation between the dependent variable (the Athens Insomnia Scale) and the independent variables. Specifically, it was found that the variables that participated in the regression model that was created and that affected the sleep quality of patients with end-stage renal failure who were undergoing dialysis were the presence of a regular caregiver and other health problems.

## 4. Discussion

This study aimed to investigate the activity level of daily life and sleep disturbances in patients with end-stage renal disease undergoing hemodialysis. The characteristics of the patients were mainly married, non-working men, who were over 60 years of age, with compulsory education and with comorbidities, and a history of medication in addition to the regimen that they received for Chronic Kidney Failure.

Our study concerned two public hemodialysis units in the region of Thessaly. The participants (n = 130) were mostly over 60 years old with usual support from a caregiver, mainly a member of the family environment, with more than five years of hemodialysis. In general, hemodialysis is a treatment approach that involves the patient staying in the hospital unit or dialysis center for several hours during the day and, therefore, is expected to affect the patient’s ability to perform any physical activity [36].

According to our results, the total score of the Barthel Index, which evaluates the activities of daily living of these patients, was found to be high, which indicates moderate dependence for the patients and, thus, the level of functionality is fairly good. Also, from this research, it was found that the total score of the Barthel Index was related to a statistically significant degree with the years that the patients had been on hemodialysis, with the hours that hemodialysis lasts, with the existence of a regular caregiver, and with the existence of another health problem or mental health issue.

Studies investigating the levels of functionality and the psycho-emotional burden of hemodialysis patients show that these are at moderate levels in these patients but are below those of the healthy population [37,38,39], and most show the level of mobility and self-care as the main dysfunctions [40,41]. Modern approaches show that systematic exercise and informing and educating patients about the benefits of exercise and how to enhance physical activity can significantly improve the physical and mental functioning of these patients [42,43].

According to our results, the patients who had informal caregivers, who did not work, and who were experiencing insomnia were those who have lower scores in Barthel’s activities of daily living. In addition, the years on dialysis were associated with higher scores in the Barthel Index. Several studies have associated the activities of daily living with various clinical and demographic characteristics. Most of them have reported that single patients with comorbidities reported poor functional statuses and decreased activities of daily living. On the other hand, we observed the paradox of the positive association of ADL and the years on dialysis. This finding is opposed to similar studies that report that many years on dialysis are associated with a poor functional status [44,45]. Our result can partially be explained by the fact that the patients were in the early years of dialysis, were married, and thus had a good support system [46].

Regarding the total score of the Athens Insomnia Scale, this indicated a mild problem with sleep for the patients, thus few difficulties in sleep characteristics, such as the sleep onset, awakenings, total duration, sleep quality, daytime sleepiness, and daytime functioning and mood [33,34]. The total score of the Athens Insomnia Scale was found to be related to a statistically significant degree with the age of the patients, their marital status, the years and the hours of hemodialysis, the presence of a regular caregiver, and the existence of any other health or mental health problem.

The correlation between the daily activity levels of the patients and their sleep quality, in our study, was negative (r = − 0.467/Sig. (2 − tailed) < 0.001) and showed that as the level of physical activity of patients with end-stage renal failure undergoing hemodialysis increases, sleep problems decrease.

The findings of the present research agree with those of the scientific literature, according to which, the association of hemodialysis with poor sleep quality in patients is recorded [47], while the sleep disorders that appear in hemodialysis patients are mainly due to the stress that they experience due to the disease and the treatment approach [36]. Insomnia in these patients can lead to increased fatigue and a poor quality of life, while at the same time, depression, immunosuppression, and cardiovascular disorders can occur. [48] These findings are consistent with our study; although the tools of the various studies differ, they seem to converge on the conclusion that patients under hemodialysis show poor sleep quality and disturbances during the day and night [49,50].

Regarding sleep disorders, although the relevant literature reports insomnia as the most important disorder [51], our study highlighted functional capacity, mood, and well-being during the day as more important problems. It should be pointed out at this point that sleep disturbances are often observed in hemodialysis patients, which, apart from being related to hemodialysis, may also be related to factors such as the advanced age of the patients, the long duration of hemodialysis, increased creatinine values in blood serum and uremia, medication, hypothyroidism, etc. [52,53].

### Limitations

Some limitations that could be mentioned are the small sample size, which was limited to a certain geographical area, and the limited time of the survey’s conduction, which both limit the generalizability of the findings and the ability to draw conclusions that can be applied to the entire population of these patients. Another point that gives some limitations to the findings of our study is the non-evaluation of biomarkers and the absence of a laboratory investigation of the factors that affect both daily activity and sleep. Finally, another limitation was that we evaluated only insomnia and no other sleep disturbances, such as restless leg syndrome and sleep apnea.

## 5. Conclusions

People with chronic kidney disease undergoing hemodialysis have a low level of physical activity and often experience sleep disturbances, creating a vicious cycle in their daily practice and burdening their daily life and areas of their physical and mental health. It is important that such characteristics are identified, and that targeted intervention is designed so that these patients can improve their lives with simple practices. Further studies and analyses are required regarding the multifactorial justification of insomnia and the impairment of daily activity in these patients. Furthermore, the observation of the phenomenon, through taking a complete history and with the help of the tools provided by the scientific community, will contribute to the early diagnosis of such conditions and the activation of care programs that will help the patient.

## Figures and Tables

**Table 1:** The sociodemographic characteristics of the sample.

		Frequency	%
Gender	Male	78	60.0
	Female	52	40.0
Age			
	Up to 30 years old	2	1.5
	31–40 years old	4	3.1
	41–50 years old	8	6.2
	51–60 years old	20	15.4
	Over 60 years old	96	73.8
Education level			
	Compulsory education	105	80.8
	Secondary education	18	13.8
	Higher education	7	5.4
Place of residence			
	Urban area	57	43.8
	Semi-urban area	35	26.9
	Rural area	38	29.2
Marital status			
	Single	27	20.8
	Married	74	56.9
	Divorced	5	3.8
	Widowed	24	18.5
Working condition			
	Working	20	15.5
	Non-employee	109	84.5
Years of illness			
	Up to 5	56	43.7
	6–10	50	39.1
	11–15	11	8.6
	16–20	6	4.7
	Over 20	5	3.9
Years of dialysis			
	Up to 5	70	53.8
	6–10	42	32.3
	11–15	10	7.7
	16–20	5	3.8
	Over 20	3	2.3
Duration of hemodialysis			
	Less than 2 h	1	0.8
	2–3 h	7	5.5
	3–4 h	94	73.4
	Over 4 h	26	20.3
Regular caregiver			
	None	25	19.2
	Family member	98	75.4
	Health professional	7	5.4
Additional health problem	Yes	99	76.9
	No	31	23.1
Mental health problem	Yes	17	13.1
	No	113	86.9

**Table 2:** The results of the multiple linear regression model (stepwise method), with the dependent variable being the Barthel Index total score and the independent variables being insomnia and the patients’ demographic and clinical characteristics (N = 130).

						95% CI	
	Unstandardized	Standard Error	Standardized	t	*p*	Lower	Upper
(Intercept)	165.467	12.361		13.387	<0.001	140.998	189.936
No caregiver reference category							
Existence of caregiver	−25.524	4.632	−0.385	−5.510	<0.001	−34.694	−16.354
Athens Insomnia Scale	−2.241	0.471	−0.329	−4.759	<0.001	−3.173	−1.309
Years in dialysis	10.012	2.338	0.282	4.282	<0.001	5.383	14.641
Working yes reference category							
	−24.848	5.807	−0.283	−4.279	<0.001	−36.343	−13.352
F(4,122) = 29.688, *p* < 0.001, R^2^ = 49.3%							

**Table 3:** The results of the multiple linear regression model (stepwise method), with the dependent variable being the Athens Insomnia Scale score and the independent variables being the patients’ demographic and clinical characteristics (N = 130).

						95% CI	
Coefficients	Unstandardized	Standard Error	Standardized	t	*p*	Lower	Upper
(Intercept)	6.650	2.063		3.224	0.002	2.568	10.732
No additional health problem reference category							
Existence of an additional problem	−3.768	0.902	−0.335	−4.175	<0.001	−5.554	−1.982
No caregiver reference category							
Existence of caregiver	2.702	0.788	0.275	3.429	<0.001	1.143	4.262
F(2,126) = 18.918, *p* < 0.001, R^2^ = 21.9%							

## Data Availability

Data supporting this study are available from the corresponding author upon reasonable request.

## Supplementary Materials

The following supporting information can be downloaded at https://www.mdpi.com/article/10.3390/healthcare12212115/s1, Supplementary file. Steps and tables of regression analysis.
