# Supplementary material for: Assessing Daily Function and Sleep Disorders in Hemodialysis Patients with End-Stage Renal Disease
Source: Healthcare (Basel). 2024 Oct 23;12(21):2115. doi: 10.3390/healthcare12212115 (PMC11545313; doi:10.3390/healthcare12212115)
Supplement: Supplementary file 1 [file healthcare-12-02115-s001.zip › healthcare-3201906-supplementary.pdf]

Table. Barthel IndexDescriptive statistics.

	Minimum	Maximum	Mean	Standard deviation
Feeding	0.00	10.00	6.53	3.77
Body Wash	0.00	5.00	3.57	2.26
External Appearance Care	0.00	5.00	3.57	2.26
Clothing - Footwear	0.00	10.00	6.76	3.77
Bowel Control	0.00	10.00	8.23	2.77
Bladder Control	0.00	10.00	7.74	3.38
Toilet Hygiene	0.00	10.00	6.88	3.64
Transportation (From Bed To Wheelchair and Back)	0.00	15.00	11.11	5.06
Motion (on a Flat Surface)	0.00	15.00	11.57	4.90
Stairs	0.00	10.00	6.88	3.80
Barthel index total score	0.00	100.00	72.90	32.13

Table. Athens Insomnia Scale Descriptive statistics.

	Minimum	maximum	mean	Standard deviation
Sleep Onset (time you need to fall asleep after lights out)	0.00	3.00	1.02	0.73
Awakening in the Middle of the Night	0.00	2.00	0.73	0.77
Final Wake up Earlier than Desired	0.00	2.00	0.68	0.68
Total Sleep Duration	0.00	2.00	0.64	0.75
Overall Sleep Quality (regardless of sleep duration)	0.00	2.00	0.76	0.74
Feeling of Wellness During the Day	0.00	3.00	1.12	0.86
Functioning (Physical and Mental) During the Day	0.00	3.00	1.15	0.88
Sleepiness During the Day	0.00	3.00	0.892	0.83
**Total score Athens Insomnia Scale**	0.00	20.00	7.01	4.72

ANOVA[a]

Model		Sum of Squares	df	Mean Square	F	Sig.
1	Regression	35391,189	1	35391,189	46,298	,000[b]
	Residual	97082,408	127	764,428		
	Total	132473,597	128			
2	Regression	47299,581	2	23649,790	34,986	,000[c]
	Residual	85174,016	126	675,984		
	Total	132473,597	128			
3	Regression	55179,416	3	18393,139	29,745	,000[d]
	Residual	77294,181	125	618,353		
	Total	132473,597	128			
4	Regression	65068,210	4	16267,052	29,688	,000[e]
	Residual	67405,387	124	543,592		
	Total	132473,597	128			

a. Dependent Variable: Barthel

b. Predictors: (Constant), Caregiver

c. Predictors: (Constant), Caregiver , Insomnia

d. Predictors: (Constant), Caregiver , Insomnia , Working

e. Predictors: (Constant), Caregiver , Insomnia , Working , Years in Dialysis

Coefficients[a]

Model		Unstandardized Coefficients		Standardized Coefficients	t	Sig.	95,0% Confidence Interval for B	
		B	Std. Error	Beta			Lower Bound	Upper Bound
1	(Constant)	137,143	9,780		14,022	,000	117,790	156,496
	Caregiver	-34,644	5,091	-,517	-6,804	,000	-44,719	-24,568
2	(Constant)	138,499	9,203		15,050	,000	120,287	156,711
	Caregiver	-27,064	5,117	-,404	-5,289	,000	-37,191	-16,938
	Insomnia	-2,189	,521	-,320	-4,197	,000	-3,221	-1,157
3	(Constant)	173,226	13,119		13,204	,000	147,262	199,190

	Caregiver	-24,308	4,955	-,363	-4,906	,000	-34,114	-14,502
	AIS	-2,076	,500	-,304	-4,155	,000	-3,066	-1,087
	Εργασιακή_κατάσταση	-22,032	6,172	-,249	-3,570	,001	-34,246	-9,817
4	(Constant)	165.467	12.361		13.387	.000	140.998	189.936
	Caregiver	-25.524	4.632	-.385	-5.510	.000	-34.694	-16.354
	Insomnia	-2.241	.471	-.329	-4.759	.000	-3.173	-1.309
	Years in Dialysis	10.012	2.338	.282	4.282	000	5.383	14.641
	Working	-24.848	5.807	-.283	-4.279	.000	-36.343	-13.352

a. Dependent Variable: Barthel

ANOVA[a]

Model		Sum of Squares	df	Mean Square	F	Sig.
1	Regression	451,959	1	451,959	24,041	,000[b]
	Residual	2387,544	127	18,800		
	Total	2839,504	128			
2	Regression	655,762	2	327,881	18,918	,000[c]
	Residual	2183,742	126	17,331		
	Total	2839,504	128			

a. Dependent Variable: Insomnia

b. Predictors: (Constant), Additional Health Problem

c. Predictors: (Constant), Additional Health Problem, Caver giver

Coefficients[a]

Model		Unstandardized Coefficients		Standardized Coefficients	t	Sig.	95,0% Confidence Interval for B	
		B	Std. Error	Beta			Lower Bound	Upper Bound
1	(Constant)	12,554	1,183		10,609	,000	10,212	14,895
	Additional Health Problem	-4,484	,914	-,399	-4,903	,000	-6,293	-2,674
2	(Constant)	6,650	2,063		3,224	,002	2,568	10,732
	Additional Health Problem	-3,768	,902	-,335	-4,175	,000	-5,554	-1,982
	Caver giver	2,702	,788	,275	3,429	,001	1,143	4,262

a. Dependent Variable: AIS
